# High Speed, Localized Multi-Point Strain Measurements on a Containment Vessel at 1.7 MHz Using Swept-Wavelength Laser-Interrogated Fiber Bragg Gratings

**DOI:** 10.3390/s20205935

**Published:** 2020-10-20

**Authors:** Steve Gilbertson, Mark Pickrell, Dario Castano, Gary Salazar, Tom Beery, Samuel Stone, Joshem Gibson

**Affiliations:** 1 Los Alamos National Laboratory, DARHT Experiments and Diagnostics, MS P940, Los Alamos, NM 87545, USA; mpickrell@lanl.gov (M.P.); gps@lanl.gov (G.S.); tbeery@lanl.gov (T.B.); 2 Los Alamos National Laboratory, Advanced Engineering Analysis, MS A142, Los Alamos, NM 87545, USA; castano@lanl.gov; 3 Lawrence Livermore National Laboratory, Environmental Test Group, L-125, Livermore, CA 94550, USA; stone58@llnl.gov; 4 Los Alamos National Laboratory, Dynamic Structure Design and Engineering, MS P942, Los Alamos, NM 87545, USA; gibson@lanl.gov

**Keywords:** fiber Bragg grating, fiber sensing, high-speed interrogation, dynamic strain

## Abstract

Dynamic elastic strain in ~1.8 and 1.0 m diameter containment vessels containing a high explosive detonation was measured using an array of fiber Bragg gratings. The all-optical method, called real-time localized strain measurement, recorded the strain for 10 ms after detonation with additional measurements being sequentially made at a rate of 1.7 MHz. A swept wavelength laser source provided the repetition rate necessary for such high-speed measurements while also providing enough signal strength and bandwidth to simultaneously measure 8 or more unique points on the vessel’s surface. The data presented here arethen compared with additional diagnostics consisting of a fast spectral interferometer and an optical backscatter reflectometer to show a comparison between the local and global changes in the vessel strain, both dynamically and statically to further characterize the performance of the localized strain measurement. The results are also compared with electrical resistive strain gauges and finite element analysis simulations.

## 1. Introduction

Fiber Bragg gratings (FBG) are routinely used for making static and dynamic strain and temperature measurements up to the kHz range [1,2,3]. Significant improvements have also been made to FBGs to improve strength and versatility [4,5,6] while also improving sensitivity [7,8]. Such measurements have been used in industry for many years, for example, in testing strain in airplanes [9] and bridges [10] or automotive traffic [11]. These relatively slow measurements are able to be made with off-the-shelf spectrometers and software packages to achieve readouts of up to a few kHz [12]. A light source illuminates a grating and the reflected light is recorded by a simple spectrometer.New measurements are made as fast as the spectrometer can read out the data, which is sufficient for measuring, for example, strain induced in bridges as cars drive over.

**Figure 1:**
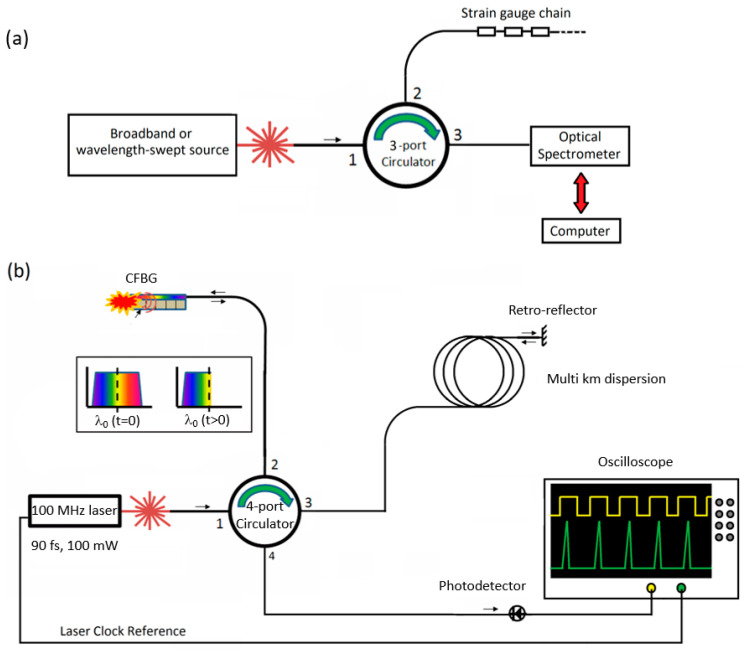
Examples of strain measurements at: (**a**) kHz repetition rates with Bragg gratings and (**b**) MHz repetition rates with chirped Bragg gratings.

On the other end of the scale, destructive testing of high explosives (HE) can also be characterized using chirped FBGs. These have been demonstrated at 100 MHz repetition rates for measuring the detonation wavefront and even some strain in the detonation process [13,14]. These experiments are often very fast (few 10s of microseconds) while also having such high pressures that the gratings are sometimes cleanly destroyed in the experiment with no measurable strain behavior.While the technology for this work is well developed, it is cost prohibitive to many labs interested in non-destructive testing.Indeed, the high bandwidth scopes alone cost well over $100K while only providing recording times of up to 2 ms. Additionally, such high repetition rates in the light source limit the number of samples that can be made in a single measurement.For example, a 50 GS/s scope measuring data from a grating interrogated by a 100 MHz laser will only have 500 points in each measurement [13]. This is a sacrifice of fine resolution for fast recording speeds in order to achieve these state-of-the-art measurements. Figure 1 shows a schematic emphasizing the differences in complexity between these 2 methods.

**Figure 2:**
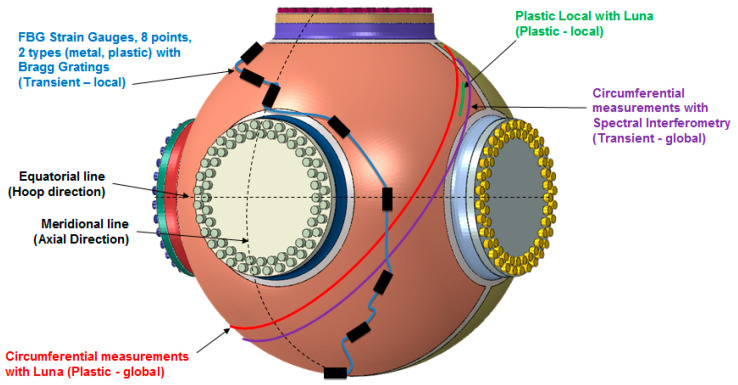
A typical explosive containment vessel used at Los Alamos National Lab. An example suite of diagnostics as discussed in this paper are indicated on the vessel surface.

Between these two extremes is a range of measurement rates of concern to safety qualifications.Rates from 1 kHz up to a few MHz are necessary for characterizing the peak and average strain in a high explosive containment vessel.These vessels are used at Department of Energy and Department of Defense national labs as well as many other areas and act as a first line of defense to the co-located workers and the general public [15]. Figure 2 shows a typical vessel used for containing an HE experiment at Los Alamos National Lab. The diagnostic suite described in this manuscript is also included in the figure.

The vessels are usually 0.9 to 1.8 m in diameter and made with high-strength low alloy (HSLA-100) steel nominally 2.54 cm thick for the 0.9 vessels and 6.4 cm thick for the 1.8 m vessels. The vessels also have access ports in order to insert experiments and other diagnostics or to act as windows for radiographic measurements to be made during the experiment. These ports increase the complexity of the strain behavior where the ports intersect with the spherical shape of the vessel.

The vessels are specifically designed to contain the pressures expected during the explosive test with calculations of the strain performed using a finite element analysis (FEA) package [16,17]. With such rigorous engineering qualifications in place, the vessels are quite expensive, exceeding $1 million each for the 0.9 m vessels and $2 million for the 1.8 m vessels. The vessels are pulled from service after several tests in order to ensure that they are not compromised during a dynamic experiment.Due to this balance between safety and economics, a suite of diagnostics capable of characterizing the vessel performance will ensure the vessels are not pulled from service too late that they are a safety risk, but also not too early that they become cost prohibitive to replace.Besides the cost of the vessel, the experiments themselves represent a multi-million dollar test of a high explosive system with specific diagnostics chosen many months in advance.

Historically, containment vessel strain has been measured on a case-by-case basis using resistive strain gauges placed in locations that can be directly compared with FEA results [18,19]. While the results have often shown agreement between experiment and calculation, other times, the results are not clear. The data areoften noisy due to additional electromagnetic or radiofrequency signals being picked up by the wires, which can act as antennas readily receiving signals between the locations where the data arerecorded and where the gauges are fielded. This noise can be several kHz, which is well above the frequencies predicted. Unfortunately, spurious strain signals cannot be fully ruled out, especially in a fast dynamic test with a high impulse from the detonation. Additionally, the gauges can break through the epoxy adhering them to the vessel surface if the impulse on the vessel is high enough. Finally, the strain measurements are localized to a few select points on the vessel, meaning the global elastic or plastic strain in the vessel is not fully characterized.

**Figure 3:**
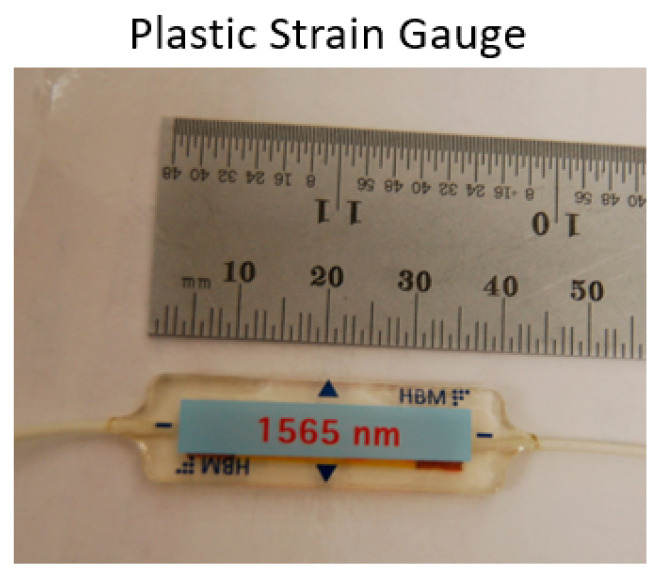
Optical light-weight plasticstrain gauges tested in our first experiments.

These limitations can be overcome through the use of optical methods for strain measurements. An all-optical path between the diagnostic and the recording location is impervious to electrical noise. The light weight nature of FBGs and fiber optics in general means the diagnostics are far more likely to stay adhered to the surface during the experiment. Because the gauges are light-weight, they are a bit more fragile than electrical gauges, although damage to the gauges has thus far been successfully mitigated through increased care in handling the vessels. Figure 3 shows an example of the optical gauges fielded in these experiments.

In this paper, the results from FBG gauges fielded on a recent 1.8 m diameter vessel during a high explosive test (hydrotest 3685) at Los Alamos National Lab will be shown. The 1.8 m diameter vessels are always used for the full scale hydrotests as they are capable of withstanding the HE pressures and holding blast mitigation inside the vessel. The diagnostics fielded were a set of robust light weight plastic gauges (HBM OL). Another option was a set of rubberized robust weldable gauges mounted onto steel plates (HBM OL-WA). For safety reasons, we were unable to weld these to the surface and so they were instead epoxied into place as a test. These gauges were much heavier than the plastic type and it was predicted they would break through the epoxy layer holding them onto the surface, which was generally found to be true. The measurement technique uses a swept wavelength laser to sequentially interrogate a set of 8 gauges placed on the vessel and at a repetition rate of ~1.7 MHz. This is sufficiently fast to oversample the strain data for accurate mapping of sound wave ringing in the vessel walls. This method can rule out or confirm fast strain spikes often seen in resistive strain gauge data as electrical noise since the spurious noise signals are typically well below the sample rate of the swept wavelength light source. As these are strictly localized measurements, two additional optical diagnostics, consisting of an optical backscatter reflectometer for static global plastic measurements and a spectral displacement interferometer for dynamic global elastic strain measurements will be compared to the localized data for a more complete picture of the vessel performance. No additional diagnostics were fielded on the vessel for hydrotest 3685 so the diagnostics had full reign over all available space on the vessel surface. This allowed us to add global strain measurements coving the full circumference of the vessel to compare with local strain measurements. The FBG data will also be compared directly to a set of resistive strain gauges from a recent overpressure test experiment on a 0.9 m diameter vessel as a comparison with the legacy diagnostics. The smaller vessel is used for testing diagnostic feedthroughs with a small HE charge. As long as the feedthroughs do not fail under an HE load, the experiment is considered a success. This smaller diameter vessel was planned to have electrical diagnostics to compare the strain experienced by the vessel with legacy data. The localized FBG method was fielded near the electrical gauges wherever possible. Due to the nature of the gauges, the circumferential diagnostics for measuring global strain were not fielded on the 0.9 m vessel. Finally, the results will also be compared with FEA calculations throughout the paper.

## 2. Materials and Methods

### 2.1. Real Time Localized Strain (RTLS) Measurements

**Figure 4:**
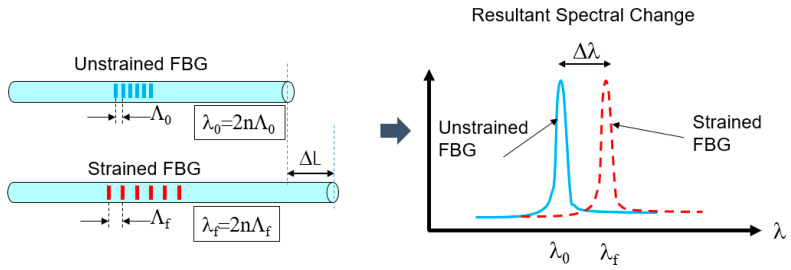
(**a**) Representation of a Bragg grating periodicity change when being strained. (**b**) Reflected spectrum of a strained and unstrained grating.

FBGs are sensitive to strain and temperature and have been used for many years [1,2,3]. The process for manufacturing the gauge is to write a periodic index change into a single mode fiber which can be done uniformly or non-uniformly [20]. The index change periodicity determines the reflected wavelength, λ, through the relation λ = 2nΛ, where Λ is the periodicity, and n is the refractive index of the fiber. By stretching or compressing the fiber, the grating periodicity increases or decreases proportionally, thereby changing the reflected wavelength linearly with the change in length. Figure 4 shows a schematic of this.

Once the wavelength changes, the strain and temperature change can be calculated from the equation:

(1) $$\frac{\Delta \lambda }{\lambda }=k\varepsilon +\left(\alpha_{\Lambda }+\alpha_{n}\right)\Delta T.$$

In Equation (1), Δ*λ* is the wavelength change, *λ* is the center wavelength of the gauge, *k* is the so called “k-factor” which is 1-p, where p is the strain-optic coefficient for the fiber and is typically ~0.21 for the optical fiber used in our experiments, *α_Λ_* is the thermal expansion coefficient of the fiber, and *α_n_* is the thermo-optic coefficient. For our fast dynamic experiments, Δ*T* changes very slowly in comparison and is basically zero. This means the majority of our wavelength change comes from the force induced strain in the vessel. The strain, *ε*, in Equation (1) is the standard value of ΔL/L.

**Figure 5:**
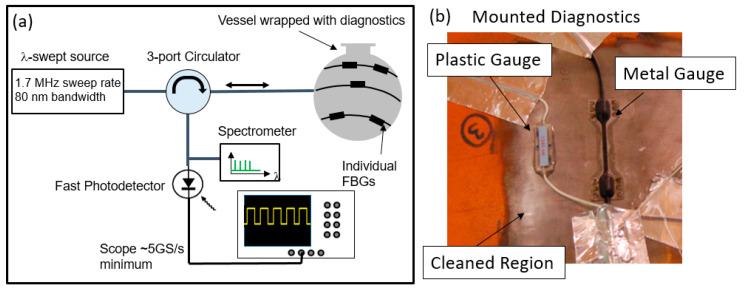
(**a**) Schematic representation of the real time localized strain(RTLS) method for measuring localized dynamic strain on a containment vessel. (**b**) Plastic and metal optical gauges mounted to a cleaned surface of the vessel.

The method for measuring the strain on a vessel is the real-time localized strain (RTLS) system. A block diagram of the technique is shown in Figure 5a. The RTLS technique is presented as an alternative for resistive strain gauges. The remainder of the paper following this section will be alternative methods for verifying the response of the optical method.

The light source used was an Optores Fourier domain mode-locked (FDML) laser [21,22]. The model used in our experiments had a sweep rate of ~1.7 MHz and a bandwidth of 80 nm centered at 1550 nm. The linewidth of the source is less than 30 picometers and the average power is ~100 mW. The laser also provides a clock signal synched to the start of sweep. The model we use creates 3 identical copies of the sweep, which are consecutively delayed and superimposed to give 4 replicas of each sweep with each start-of-sweep of the laser. This means the clock signal frequency, while synched to the start of sweep, is actually a quarter of the frequency of sweeps that the laser provides. This has to be taken into consideration for the data analysis. Finally, the stability of the laser was measured to be less than 180 ps after 10,000 sweeps, which is much less than the 2.36 microsecond sweep period of the laser. This stability result is also dominated by the trigger jitter of the detector so the laser stability may be even better than this.

The output from the laser is sent to a 3 port circulator and directed to a chain of FBG strain sensors placed on the device under test (DUT), which in this case is a confinement vessel. Each sensor reflects one particular wavelength which is then sent back to the circulator. Due to the sweeping nature of the laser, each gauge is interrogated at a unique time with respect to the start of sweep that does not change in a static measurement. The sequential series of reflections is then sent to a 99:1 splitter with 1% of the light sent to a spectrometer for calibration purposes. Each peak represents one gauge placed on the DUT. The remaining 99% of the light is sent to a fast photodetector, in this case, an Optilab PR-23-M 23 GHz optical detector. The output of the detector is recorded on a digital oscilloscope. In order to record fast temporal changes in the strain on the vessel, a 4 GHz scope capable of measuring up to 25 GS/s was used. The extended memory option allowing up to 125 M points per channel was also required and even with this, the sample rate had to be reduced to 12.5 GS/s in order to record for 10 ms. The high sample rate allows measurements to be made with a frequency limited by the sweep rate of the laser source. In this case, 1.6 MHz which is over 300 times more measurements per unit time than standard off-the-shelf spectrometer-based measurement techniques.

**Figure 6:**
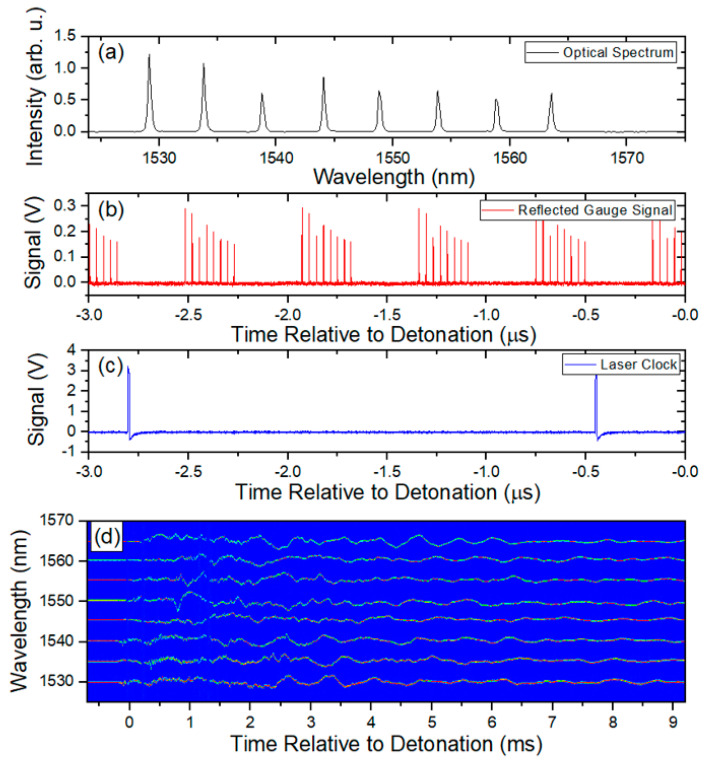
Representative data accumulated with the RTLS method. (**a**) Typical reflected spectrum from a chain of optical strain gauges. (**b**) Reflected response of the grating as measured as a function of time. Each set of 8 peaks represents a single measurement of all the gratings in a chain made by one sweep of the laser. (**c**) Recorded clock signal of the laser. Since the laser makes 3 replicas of the laser sweep, each peak is synched with the start of sweep of the laser but occurs every 4th pulse. (**d**) Example of the 2D array of strains measured with each chain of optical strain gauges. Each horizontal signal represents the temporal history of the strain on the vessel at one particular location during the first 10 ms after detonation.

The chain of gauges used for the localized measurements is a custom configured design manufactured by HBM. The chain was chosen to include 8 wavelengths that fit within the bandwidth of our laser source. For our proof-of-concept experiments, we focused on one type of FBG chains. The gauge is a light weight plastic-encased FBG. The gauge is particularly flexible, which aids in the installation of the gauges on a curved surface. The gauges can be prepared with any amount of optical fiber between them, thereby allowing the gauges to be placed at any location and in any orientation on the contour of the vessel. To attach the gauges, we first had the vessel surface prepared by removing the paint with a grinder. The bare metal was then scuffed with 400 grit sandpaper. Next, the surface was cleaned with phosphoric acid followed by a neutralizer. Finally, the gauges were attached to the surface with a thin layer of two part 5 min epoxy. The gauges were held in place with a Teflon cushion until the epoxy was set. An image of the gauges attached to a sample vessel is shown in Figure 5b. The gauges employ a single mode fiber etched with a grating. The maximum strain measurable for both types of gauge is +/−10,000 µε and the peak reflectivity at the specified wavelength is 15%. The linewidth of a single FBG is specified at 0.13 nm and the FBG inside the gauge is 5 mm long. A sample spectrum for a series of 8 gratings is shown in Figure 6a.

A second type of weldable FBG with a rugged rubberized coating on the fiber optic was also tested. Due to safety considerations, we were not able to weld the gauges to the containment vessel, so they were attached using the same epoxy method of the plastic gauges. Due to the increased mass of the weldable type, it was expected that they would have a larger chance of breaking through the epoxy that was adhering them to the surface at any time during the dynamic response of the vessel walls following the HE detonation. These gauges generally failed for these reasons.

The peak wavelengths were chosen to coincide with our laser source bandwidth. We also designed the chain with 5 nm separation between each peak wavelength, corresponding to over 4000 µε, which exceeds the peak strain often expected in the vessels. This helps ensure the peaks do not cross during the experiment for ease in data analysis.

To form an accurate measurement of the strain, the data must be first converted from the time axis as it is recorded on the scope to the spectral domain. The first step is to parse the data according to the start of each sweep. This is done by recording both the data and the clock signal during the experiment. The data shown in Figure 6b,c show the raw data and the clock signal, respectively. The clock period is then extracted and divided by 4 since the laser source we use creates 4 sweeps for each clock pulse. Each raw data sweep is then extracted using the quarter clock period with the time of each start of sweep recorded. The time axis for each sweep is relabeled from t0 to N, where N is the total number of samples multiplied by the time step in a single sweep. In the case of our light source, we record ~7800 samples for each sweep. With a 12.5 GS/s sample rate, our time step is 80 ps, giving a total time window for each sweep of nearly 625 ns. Next, the new 0 to N time axis must be converted to wavelength. A sample calibration spectrum as seen in Figure 6a is recorded shortly before each experiment. This is done as close to the experiment as possible to ensure the temperature of the vessel does not vary much from before to after the experiment. A set of thermocouples is also fielded on the vessel, which allow for independent verification of the temperature. From the sample spectrum, the peak of each gauge is found. A polynomial fit of the peak wavelengths versus the temporal point as recorded on the time axis of the scope is produced. This gives a fitting function that allows every temporal sweep of the laser to be converted into wavelength. Once this has been accomplished, a 2D array of each temporal sweep converted into wavelength and plotted against the start time of each sweep can be made. It should be noted that the spectrum shown in Figure 6a has a very similar profile to the temporal representations shown in Figure 6b, demonstrating that the spectral information is preserved in the measurement. Figure 6d shows a sample 2D array plotted as an image with the intensity of each peak plotted as a color. Each separate line is the wavelength change for each gauge. From this point, extracting the wavelength change for each gauge and converting into the strain using Equation (1) is straightforward.

### 2.2. Global Plastic and Elastic Deformation of Vessels

The RTLS measurements can give an overview of the localized strain at various points on the vessel. The individual measurements only record for 10 ms however, and so plastic deformation in the vessel cannot be fully deduced from the RTLS measurements alone. To further characterize the vessel and complement the local transient measurements, two additional techniques are typically fielded. These additional diagnostics give insight into the global change, both statically and dynamically, by integrating the total strain along the circumference of the vessel. 

**Figure 7:**
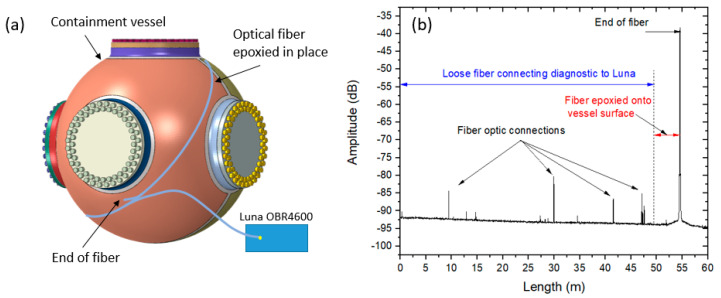
(**a**) Location of a single optical fiber placed around the circumference of a vessel for Luna measurements. The fiber has to avoid the ports of the vessel and is unable to follow a true great-circle of the vessel. (**b**) Luna optical backscatter reflectometer (OBR) trace for a fiber stretched around the vessel circumference.

The first is a simple technique using an optical backscatter reflectometer (OBR) with high resolution. The device we chose is a Luna OBR4600 [23] which has an ultimate resolution of 10 µm. The Luna device is slow compared to the detonation event of the experiment with a single scan taking a few seconds, so we treat the measurements statically. Figure 7a shows how the Luna device is employed for global measurements. First, the vessel is prepared by removing the paint and creating a surface in a manner similar to what was described for the RTLS method. Next, a fiber is attached with epoxy onto the vessel in a great circle around the vessel. A slight amount of tension is applied to the fiber during the epoxy application to ensure the fiber is tight around the vessel. A gold reflector is sometimes attached to the end of the fiber to increase the visibility of the location of the end of the fiber, although this is not really necessary due to the large dynamic range of an off-the-shelf OBR4600. From here, static measurements can be made of the fiber length that has been stretched around the vessel. Figure 7b shows a sample Luna OBR4600 dataset.

The individual peaks represent locations of fiber connections with the final peak at 55 m being the end of the fiber. As the measurement is slow, several scans can be made to show any length changes in the vessel due to temperature changes all the way up to the experiment. Typically, we scan every 15 min for an hour before and after the experiment. The global change to the circumference of the vessel includes any plastic deformation as well as temperature changes due to the detonation event that occurred during the experiment. Length changes from temperature induced expansion must be considered in the analysis of the data using complementary thermocouples fielded on the outside and inside of the vessel. Further discussion of the temperature effect will be discussed in the results section. Additionally, a shorter piece of fiber optic was attached to the vessel. Since it is not a full circumference of the vessel, a scaled measurement of the length change can be measured and compared with the full length fiber around the total vessel circumference.

**Figure 8:**
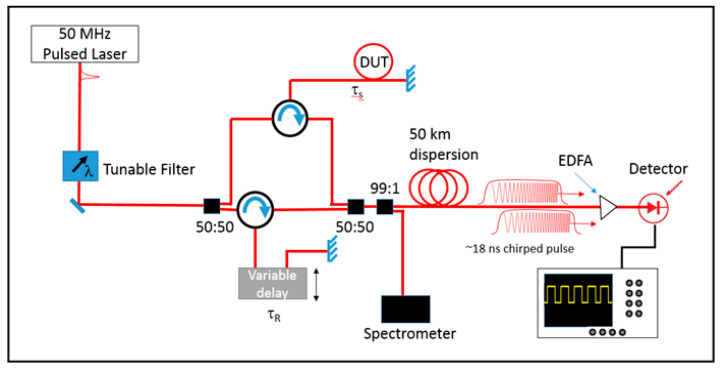
Schematic representation of the experimental setup for spectral interferometry for transient strains (SITS). DUT is device under test, EDFA is erbium doped fiber amplifier.

The second technique for global measurements makes a transient measurement in real time during the first 10 ms after the detonation event. This is an elastic measurement that can be more closely compared with the RTLS measurements. The technique is spectral interferometry for transient strain (SITS) measurements and a schematic is shown in Figure 8.

First, a laser operating at 50 MHZ with an average power of 50 mW and pulse duration of ~100 fs was sent through a tunable filter. This allowed the spectral content of the laser to be modified for either increased range in the measurement or increased resolution. Using more bandwidth reduces the range over which length measurements can be made but also increases the resolution. The laser next entered the interferometer portion where it was split into two equal replicas with a 50:50 splitter. Each half of the light was sent to a 3 port circulator. One leg of the interferometer had the light sent to the device under test (DUT). In this case, the DUT was a vessel with a single optical fiber wrapped around it. A gold reflector was attached to the end of the fiber in order to capture as much of the light as possible. The light then reflected from the gold reflector and traveled back to the circulator. The other leg of the interferometer acted as the reference and had the light traveling through a variable delay and reflecting from another gold reflector. The two legs then recombined at a second 50:50 splitter. From here, 1% of the light was split off and sent to a spectrometer for comparison purposes. The remaining 99% of the light was sent through 50 km of dispersion which, along with the optical detector, acted as a dispersive Fourier transform [24]. This allowed us to measure a representation of the spectral content of the combined signal on a fast oscilloscope. An erbium-doped fiber amplifier (EDFA) was also included for post amplification if the signal was not strong enough. The scope chosen was a 25 GHz Tektronix scope with optional high memory. This allowed record lengths of 10 ms at 50 GS/s.

**Figure 9:**
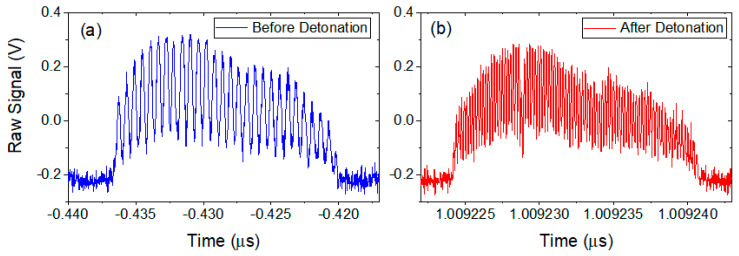
Sample spectral interferograms as recorded on an oscilloscope as a function of time for (**a**) closely spaced and (**b**) widely spaced optical pulses. The fringe visibility is beginning to be limited by the bandwidth of the scope in (**b**).

As this is a spectral measurement, interference fringes can be seen on the scope. Figure 9 shows a sample set of fringes for two different delays between the two legs of the interferometer. When the delay between the two pulses is small, the fringes are large. However, when the DUT is put under strain, the fiber is stretched and the delay between the two pulses increases, causing the fringes to become much finer.

The longitudinal resolution of the measurement is dependent on the bandwidth of the source, while the total length change that can be measured is dependent on the total bandwidth, Δf, of the recording scope and the repetition rate, f_rep_, of the light source. The resolution is given by:

(2) $$\Delta z=\frac{\lambda_{0}{}^{2}}{2\left(\Delta \lambda \right)},$$

where *λ*_0_ is the center wavelength of the source and Δ*λ* is the laser source bandwidth. The total range of the measurement is limited by when the scope can no longer resolve spectral fringes. By reducing the repetition rate of the source, an individual pulse can be further stretched so as to resolve fringes corresponding to larger separations between the signal and reference pulses without pulse to pulse overlap, although this is at the sacrifice of temporal resolution. The temporal period, T, of the fringes in the interferograms seen in Figure 9 is given by T = 2πβ_2_L/τ and the dispersion of optical fiber can be calculated from D = 2πcβ_2_/λ_0_^2^, where L is the length of the dispersion fiber in the setup, β_2_ is the group velocity dispersion in the fiber, and τ is the delay between the two pulses in the interferometer [25]. The smallest value of T, and hence the largest value of τ that can be measured is limited by the scope bandwidth, Δ*f*, and is 1/Δ*f*. Solving for τand noting that the total range R*_max_* that can be measured is cτ/n, the full range is given as:

(3) $$R_{max}=\Delta f\lambda_{0}{}^{2}DL/n,$$

where *n* is the refractive index of the fiber. The units of *DL* are (ps/nm). Equation (3) can be expressed in units of the desired resolution, Δz, using Equation (2) as R*_max_* = 2(Δz)(Δλ)(Δf)DL/n. However, multiplying the total dispersion inherent in the setup, DL, by the bandwidth of the laser pulse Δλ, yields the duration of the laser pulse after propagation through the dispersive medium. The longest value this product can be without pulse-to-pulse overlap is the laser periodicity T_L_ = 1/f_rep_.

For a desired resolution as calculated from Equation (3), the total maximum range which can be measured is therefore given by:

(4) $$R_{max}=\frac{2\left(\Delta z\right)\left(\Delta f\right)}{nf_{rep}}.$$

With a 50 MHz repetition rate and 25 GHz bandwidth scope, ranges of 3 cm from resolutions of 30 µm would be possible. Additionally, a new measurement would be made every 20 ns which allows for high precision in temporal changes of the total circumference of the vessel.

**Figure 10:**
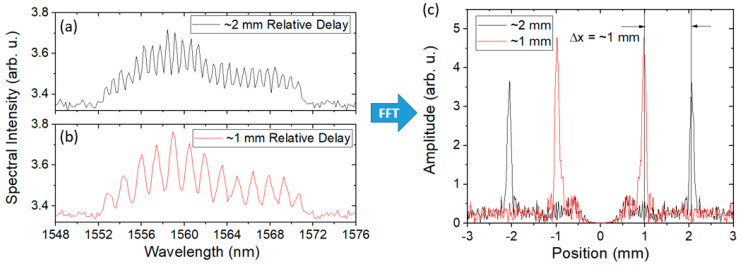
Sample spectral interferograms as recorded on a spectrometer for (**a**) 2 mm spaced and (**b**) 1 mm spaced optical pulses. (**c**) The fast Fourier transform of the results after subtracting the DC component. There are two sets of sidebands for each delay, symmetric about 0 delay.

To analyze data in SITS measurements, the time axis on the scope must be changed back into the spectral domain. A method similar to how the data is parsed and re-labeled with a wavelength axis in the RTLS measurements is first conducted on the raw SITS data recorded from the scope. The wavelength range was determined by comparison with the spectrometer measurements made before the experiment. The DC component of the signal is subtracted and a Fast Fourier Transform (FFT) is applied to every temporal measurement. The FFT yields two sidebands located at +/− the delay between the two pulses and is dependent on the fringe separation in the spectral domain. As the strain measurements are made by the length change in the vessel, the initial difference in delay from before the detonation event must be known. To accomplish this, static spectra were recorded a few minutes before the experiment began with known delays between the two legs of the interferometer. These acted as calibration spectra. Again, the DC signal was subtracted and an FFT was applied to each spectrum. Figure 10 shows a sample result of this analysis. The FFT determined the initial offset between the DUT leg and the reference leg to be 2 mm. 

When a 1 mm delay was removed from the reference leg, the sidebands showed a corresponding shift of 1 mm. A 2D image showing the location of the sideband as a function of time was made to visualize the total length change during the experiment.

For the actual experiment, care was made in the initial tuning. The zero delay point represents no fringes in the spectral domain and we chose to avoid it so that the sidebands would not cross. As it was expected that the fiber would stretch during the experiment leading to increased delay, the initial offset was adjusted such that the positive sideband would start at some pre-defined value and only move to a larger value rather than moving towards the 0 delay.

This particular diagnostic acted as a bridge between the RTLS measurements and the Luna measurements to measure the global circumferential change in the vessel during the dynamic experiment. The SITS result could be compared with the individual strain gauges to show how the total circumference relaxes dynamically or compared with the Luna measurements to show how the vessel plastically deforms.

The optical techniques listed in this section can be summarized in the following table (see Table 1). RTLS and SITS both measure dynamically the transient response of the vessel as it elastically returns to the unstrained state, but only for a limited amount of time after the start of the experiment. The short timescales of these diagnostics limit the amount of information that can be gathered due to plastic or permanent deformation in the vessel. Luna measurements are employed for static or plastic deformation in the vessels due to the slow nature of the recording method.

### 2.3. Electrical Strain Gauges

All strain gauges were installed at a vessel preparation facility prior to the movement of the vessel to the firing site. A total of 9 strain gauge rosettes were installed on the vessel exterior. One of the 9 strain gauge rosettes was used as a dummy gauge and all three gauges on that rosette were wired. This gauge acted as a dummy gauge because it was not provided with excitation power during the test, so a response seen on this gauge is electrical noise. Vishay micro-measurements CEA-06-250UR-350 strain gauges were selected based upon the vessel material, expected strain, mounting configuration, and sensitivity. This part number corresponds to a constantan grid, completely encapsulated in a polyimide rosette that has a 0.25 inch gauge length and a nominal resistance of 350 ohms. The gauges selected are also temperature compensated for steel, though this is not a necessary requirement for the dynamic strain measurement being made during this test. The gauges have a nominal gauge factor of 2.1 for grid 1 (horizontal), 2.125 for grid 2 (diagonal), and 2.1 for grid 3 (vertical).

The resistance change of a strain gauge is very small and often poses a challenge to accurately measure. While many different methods exist to measure change in resistance, a Wheatstone bridge powered by a precision voltage source is commonly used as it provides the capability to measure both static and dynamic signals. The purpose of the Wheatstone bridge is to provide a means of measuring a voltage centered at 0 V and correlating that voltage to a resistance. A balanced bridge provides a reference voltage centered at 0 V. The sense voltage can be amplified via an operational amplifier. This method is an improvement over directly measuring the gauge resistance as resolution error is reduced. Adding such complexity into a measurement system brings additional challenges regarding the uncertainty and increased introduction of noise.Accuracy can be degraded by factors such as voltage losses in the lead lines attached to the gauge. Three wire gauges used in the quarter bridge configuration minimize this gauge desensitization effect [26] as opposed to a two wire configuration. While the three wire strain gauge configuration possesses other attributes such as temperature compensation, the thermal capacitance of the vessel negates thermal dynamics. 

Nearly all the components necessary in a measurement including the operational amplifier, low pass filter, 5 MHz analog to digital converter, and precision bridge excitation are packaged into a commercially available data acquisition system. Bridge completion is external to the data acquisition system by using rack mounted precision 350 Ohm resistors. Care is taken to minimize the length of wire from the gauge to bridge completion reducing the desensitization effect. It is also desirable to minimize the length of wire before amplification. This has the potential to reduce the amount of noise that is amplified. The overpressure test required about 15 feet of wire to bridge completion and another 10 feet of wire before amplification. Both the vessel and data acquisition chassis are tied to the building electrical ground. This method introduces a potential ground loop and potential 60Hz noise but ensures components are electrically safe to touch. 

**Figure 11:**
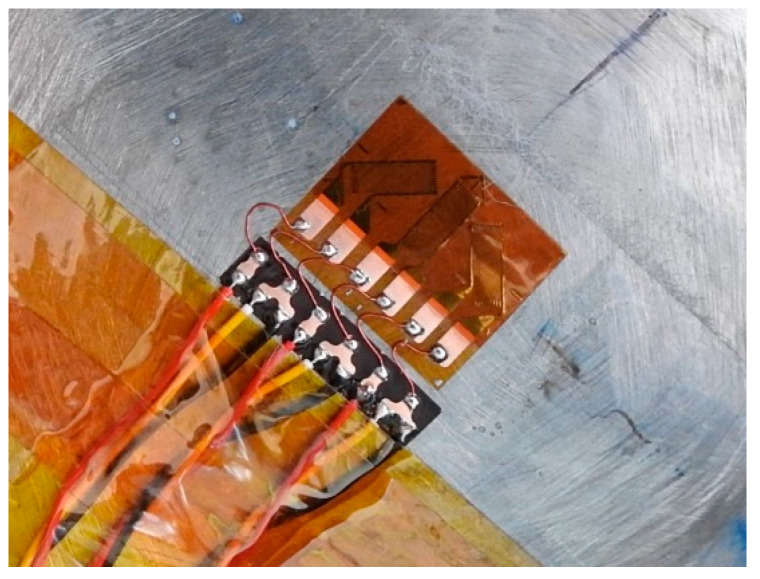
Foil strain gauge with adjacent solder tabs.

During the shot, vessel acceleration is violent enough to rip wires off solder tabs due to inertial cable loading. Through practice, Lawrence Livermore National Lab has found this effect best mitigated by using small gauge wire (30 AWG) and strain relief wire loops between the gauge and solder tabs shown in Figure 11.

Proper gauge application is also necessary to ensure intimate contact of the gauge with the surface. Vishay surface preparation recommendations are followed including paint removal with an angle grinder, directional sanding, and pre-bond surface preparations applied. Vishay M-Bond AE-10 is applied to gauges and solder tabs to bond to the vessel surface. A vacuum pad is placed over the gauge to apply pressure to the gauge and the vessel surface for a day while the bond cures. A layer of Vishay M-Coat-A polyurethane is applied to the strain gauge and the solder tabs to protect gauges and provide electrical insulation.

### 2.4. FEA Simulations

**Figure 12:**
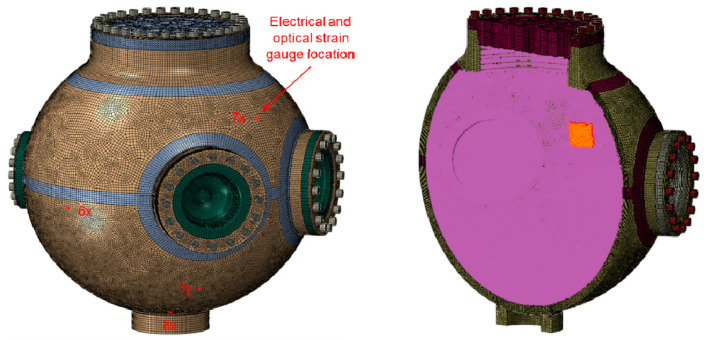
FE model of the 0.9 m vessel. Shown on the left are the elements used to compare the FE strains to the test measurements. The orange area highlighted on the cut-out on the right are element faces in the neighborhood of a tracer; the inside wall of the vessel is subjected to about 770 different pressure profiles.

Prior to an explosive test in a vessel, a hydrodynamic computation (for example, with CTH from Sandia National Laboratories) is conducted to simulate the HE blast and to calculate pressure history profiles at many tracer points located next to the internal wall of the vessel. The development of optical diagnostics capable of delivering low noise and high accuracy measurements of the strain are critical to improving the modeled vessel performance. As such, the experimental data is being used to verify or improve the FEA code rather than the code being used to explain the model. Normally we use 2D, axisymmetric models for the hydrodynamic computation, but depending on the HE setup inside the vessel, we also compute pressure profiles using 3D models. Next, with an explicit, dynamic Abaqus 3D FEA, we verify the vessel’s structural integrity by subjecting it to the internal pressures computed with the hydrodynamic software. The pressure profiles are applied to areas assembled with element faces that are close to the location of their corresponding tracer point. Figure 12 depicts the FE model of the 0.9 m vessel with a shell thickness equal to 2.82 cm that was subjected to an overpressure test. 

**Figure 13:**
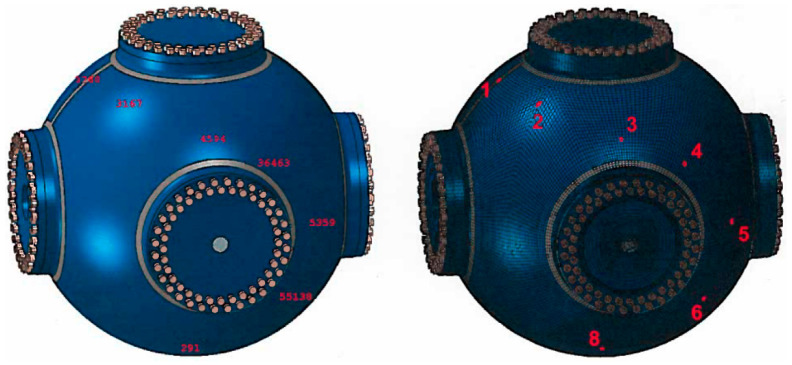
FE model of the 1.8 m vessel. The elements numbers for the FE correlation are shown on the left figure and the optical gauge number is on the right next to its corresponding element.

Highlighted on the left figure are the finite elements that were used to correlate the computed strains to the test measurements, and on the right we show an small area to which we apply a pressure profile next to a tracer. To secure the covers to the nozzles, we preload the FE model for the bolts to the actual preload for the test; for example, in the 1.8 m vessel with a 6.35 cm thick shell shown in Figure 13, the preload for each bolt (of 64 per cover) is about 245,000 N (55,000lbf). It should be noted that the bolt preload affects the FE computation. Based on our experience with the tests and FE analyses, we run the FE computation to 10.0 ms, which is when the vibration of the vessel is diminished because of the natural material damping and the friction between covers and nozzles.

Briefly, these are the sequence of events during the transient FE results of an 1.8 m confinement vessel (Figure 13) when subjected to in internal HE blast: Following the first pressure surge at the tracers on the internal wall, which might last for about 0.5 ms, the vessel’s shell expands and contracts in 1.0 ms. In the 1.8 m vessel, the heavier ports are slower to react to the initial pressure and the internal stresses from the expanding shell, but once they catch up with the dynamics of the shell, the whole vessel attains at near 2.5 ms a natural vibration. In general, when vibrating at its natural frequency of about 1300 Hz, there are locations on the vessel that reach their highest strain/stress levels. At 7.0 ms, the elastic stresses that sustained the natural vibration begin to dissipate as the results of damping and friction. Figure 13 shows the element numbers chosen for the finite element correlation in the left figure. The right figure shows the optical gauge number in the experimental FBG chain. For example, number 1 is the first gauge corresponding to the 1530 nm central wavelength and number 8 is the final gauge corresponding to the 1565 nm central wavelength.

**Figure 14:**
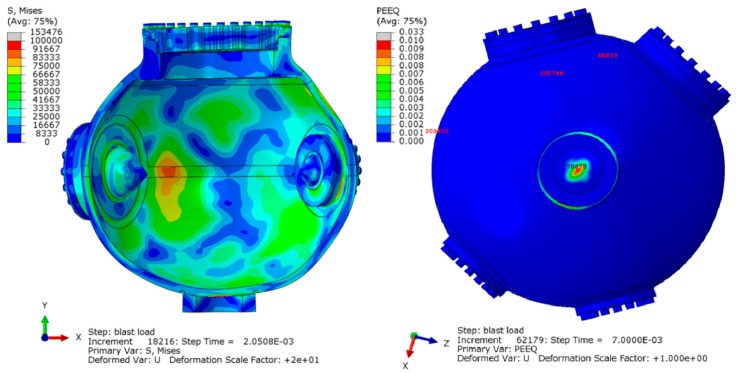
Mises stress at 2 ms, shown on a deformed cut-out, which has been scaled by a factor of 20. As shown the equivalent plastic strain (PEEQ) value is 1% plastic strain at the south pole of the vessel.

To assess the structural soundness of the vessels subjected to impulsive loading, the FE results are compared to the limits stated in Code Case 2564 of the ASME Boiler and Pressure Vessel Code, Section VIII, Division 3. A Mises stress field and the equivalent plastic strain (named PEEQ in Abaqus) are shown in Figure 14. Note that the south pole of the vessel, located on the y-axis, accumulates large plastic strains. This is because the south pole is active in most modes of vibration of the vessel, and furthermore, it is locally blasted with a high pressure. Additionally, we compute transient loads in the bolts, which should not exceed the proof load of a bolt, and the relative displacements between covers and nozzles to ensure that there will not be any outgassing. Therefore, we recognize that to comply with the structural requirements of the vessels to the large amount of HE in the tests and the inherent difficulty when computing their internal pressures and dynamic response, we must validate our integrated hydrodynamic and FEA against strain measurements obtained on the shell of the vessels. Because this is our first effort, we have not adjusted parameters for the hydrodynamic and FE computations in order to obtain better correlations. Currently, the metrics for the comparisons simply include: visuals of the FE strain vs. the optical strain measurement; the short time Fourier transform (STFT) to assess the frequency vs. time strain responses; and a FFT to assess the natural frequency of the vessels when the strains become large. Comparisons between these metrics will be shown in later sections.

## 3. Results

### 3.1. Hydrotest 3685

#### 3.1.1. RTLS Results

The first experiment where the vessel strain diagnostics were fielded was a proof-of-concept test of the strain on a containment vessel surface for Hydrotest 3685. A detonation event was staged inside a 1.8 m containment vessel at the Dual Axis Radiographic Hydrodynamic Test (DARHT) facility at Los Alamos National Lab. The experiment consisted of 8 FBG based strain gauges for use with the RTLS technique and 2 single fibers wrapped around the vessel for the Luna measurements and the SITS measurement. The combination of these three strain measurements allowed the overall vessel performance to be recorded and characterized both locally and globally.

**Figure 15:**
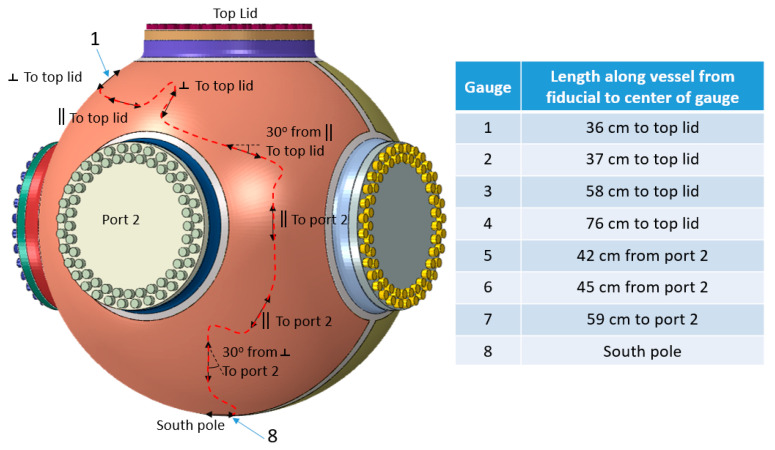
Locations and orientations of the gauges on the vessel for hydrotest 3685. The distances to a known fiducial are also shown. These maps are provided to the modeling team in order to compare the measured strain with the pre-shot predictions at these particular locations.

The RTLS measurements are the primary way to characterize the vessel performance as they can be compared with legacy experiments that had electrical gauges fielded on them. For the 3685 hydrotest, the FBG gauges were laid out as seen in Figure 15. Figure 5b shows a sample of two gauges fielded on the vessel for hydrotest 3685.

**Figure 16:**
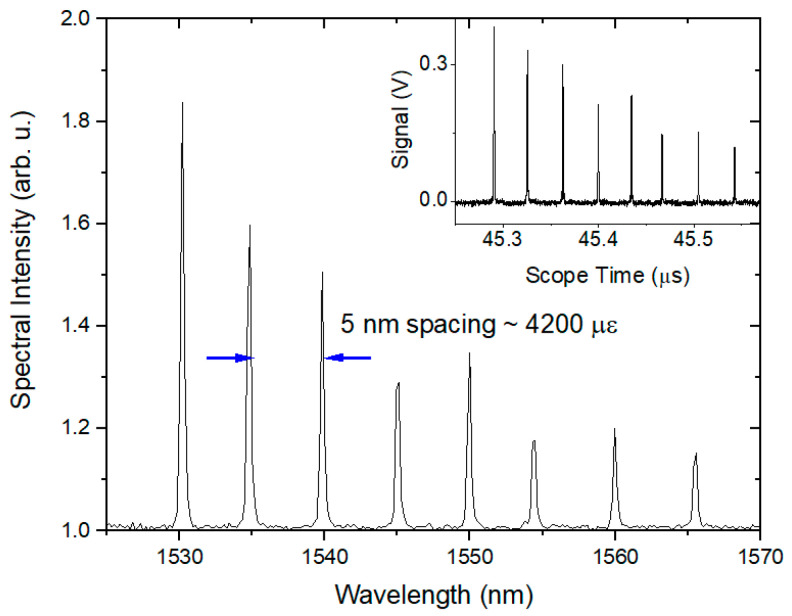
Reflected calibration spectra recorded with a spectrometer for the plastic-backed optical strain gauges. The insets show the reflected spectrum as recorded with the RTLS technique before the detonation event. In both cases, the RTLS representation qualitatively matches the spectral response very well.

For this first experiment, the gauges were positioned so that they would not interfere with other diagnostics. The mounting procedure was described in the Materials and Methods section. The FE analysis was performed with the gauge locations and orientations as measured to show the overall agreement. The gauges consisted of an FBG chain of the small light weight plastic type. Before the initiation of the detonation, sample reference spectra were saved for the chain of gauges. The results can be seen in Figure 16 for the plastic gauges.

In the static case, a single gauge can undergo 4200 µε of strain before the wavelength shift would cause the gauge data of two neighboring gauges in the chain to overlap. If the gauges moved in opposite directions, the amount of strain before overlap could be much less. Along with the measured spectra shown in Figure 16, a snapshot of the static result as measured on the oscilloscope is also shown. It is clear that the relative peak heights and spacing are preserved in the purely spectral measurement and with the swept wavelength laser reflecting from each grating in the chain. This is important for calibration purposes during the data processing portion of the experiment.

**Figure 17:**
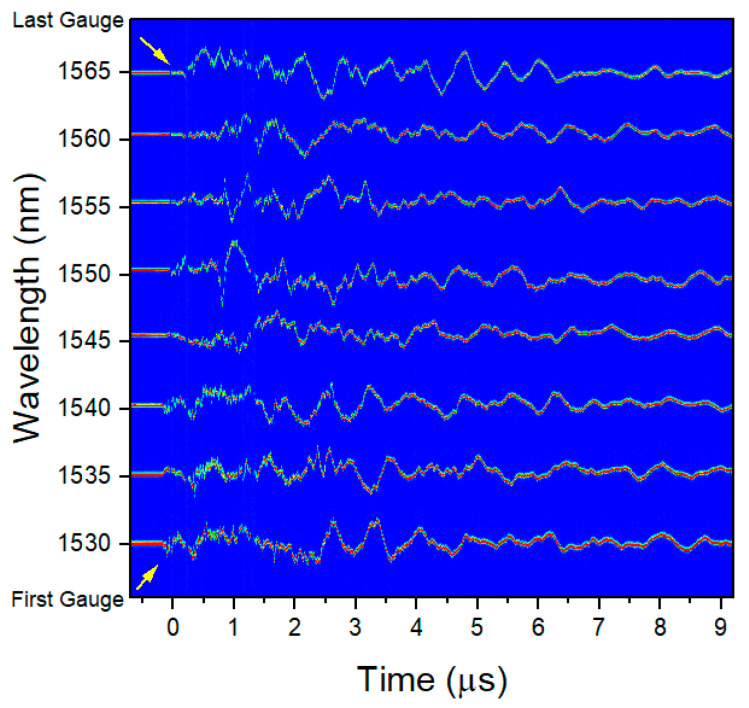
Experimental RTLS measurements for an 8 optical strain gauge chain on hydrotest 3685 for plastic-backed gauges. All 8 of the plastic-backed gauges remained on the surface. The center wavelength of each gauge is listed on the vertical axis.

After analyzing the data, 2D plots of the signal recorded in each gauge were made as shown in Figure 17. Each horizontal line is a different gauge plotted as a function of time. For the plastic gauges shown in Figure 17, 8 clean, individual signals with no crossings were recorded.

**Figure 18:**
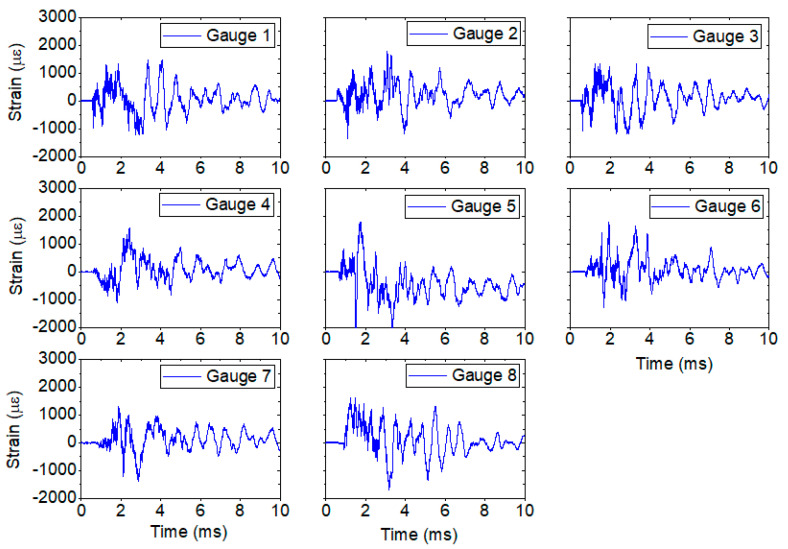
Optical strain measurements for plastic-backed strain gauges for hydrotest 3685. All results show consistent peak strains and ringing. Gauge 1 is shown at the bottom of Figure 17a and corresponds to center wavelength 1530 nm. The other gauges follow in 5 nm increment center wavelengths.

The plastic gauges yielded strain measurements at all 8 points for the 3685 hydrotest. Figure 18 shows the results for all 8 gauges. A ringing periodicity of ~0.7 ms is seen in all 8 gauges, consistent with the expected frequencies calculated in the FEA. The source of the ringing is the natural breathing of the vessel after HE detonation when the masses of the port covers are taken into consideration. Peak strains of nearly 2000 µε were seen after the initial transient from t0 to ~2.5 ms. This was well below the yield strength of the vessel and indicated that the strain at the 8 points tested did not exceed the safety requirements for the vessel.

#### 3.1.2. Luna and Spectral Interferometry for Transient Strain (SITS)

**Figure 19:**
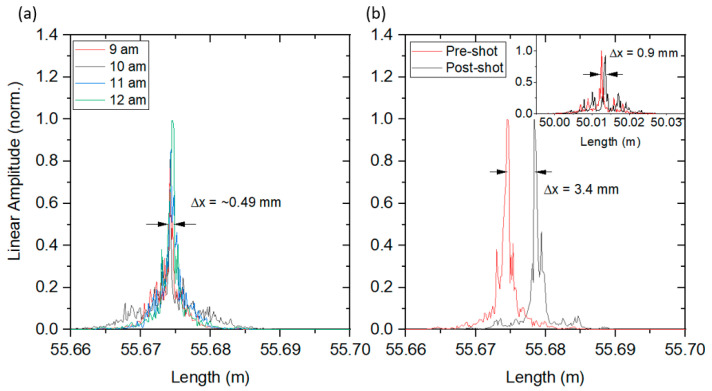
Luna OBR results for hydrotest 3685. (**a**) OBR length measurements prior to detonation in 1 h increments. The total length of the fiber varied by 0.49 mm over this time and can be attributed to the vessel thermally expanding as the temperature increase during the day. (**b**) OBR length measurement immediately after the detonation event. A total length change over the full circumference of the vessel was measured as 3.4 mm. The inset in (**b**) shows the result of a fiber 25% the length of the circumference epoxied onto the surface. The total length change was found to be 0.9 mm, which was consistent with the full circumference fiber.

The local transient measurements of the elastic strain on the vessel can be combined with the global transient and static measurements for characterization of the elastic and plastic deformation of the vessel, respectively. The vessel used for hydrotest 3685 had a diameter of 1.8 m. The circumference was 5.7 m but, due to the locations of the ports on the vessel, a true great circle around the vessel was not available. The fiber for this shot was routed around the vessel as close to a great circle as possible. For the global plastic measurements, Luna OBR4600 data wereused. For several hours prior to the start of the experiment, Luna scans were saved in 15 min intervals. Figure 19a shows the stability of the circumference of the vessel before the detonation event. A spread in the data of 490 um was observed over the course of 4 h which can easily be attributed to thermal expansion of the vessel as the temperature rises from the morning until shot time. After detonation, scans were again saved in 15 min intervals. As the vessel cooled, small changes due to thermal expansion could be recorded as well.

Only small changes were observed after the detonation event, implying the vessel either stayed at an elevated temperature for an extended period of time, was elastically deformed due to the increased pressure from the phase change of the HE products from solids to gas, or was plastically deformed. As the RTLS data did not imply plastic deformation at any of the 8 measured points, the increased circumference was probably due to pressure and temperature changes in the vessel. Figure 19b shows the total change recorded by the Luna OBR4600 from 4 min before the shot until 1 min after the shot. A total length change of 3.4 mm was recorded around the full vessel, which did not change over the course of an hour. Additionally, the inset of Figure 19b shows the result of a 1.4 m fiber also attached to the vessel. This fiber was ~25% of the length of the total circumference fiber and so a corresponding reduced length change was expected. The results were consistent with a 0.9 mm length change as measured in the shorter fiber.

**Figure 20:**
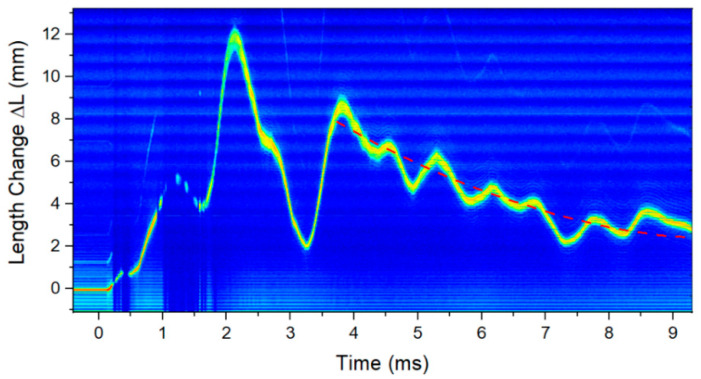
SITS result for the total circumferential change of the vessel during the first 10 ms after the detonation event for hydrotest 3685. Peak values of over 1 cm were seen and the overall result decayed back to an increased circumference by the end of the 10 ms recording time.

The dynamic change in the total circumference was also recorded using the SITS technique. The total length change of the vessel was calculated for each pulse of the laser, resulting in 50,0000 individual length measurements for the 10 ms time window that data were recorded. Figure 20 shows the 2D image of the sideband plotted as a function of time.

The vertical axis has been calibrated as the change in length of the total fiber and the axis has been shifted so that 0 change occurs prior to t0. This is the original offset in delay the two legs of the interferometer were set to. The overall effect is consistent with the RTLS measurements. A peak value is found around 2 ms which was seen in many of the individual gauges fielded with the RTLS technique. Additionally, the minimum in the data was found around 3 ms when the vessel quickly relaxes after the initial shock from the detonation. Since this is a total length change, the results in Figure 20 show that the vessel circumference increased by over 1.2 cm at the peak. Dividing this by the total vessel circumference yields a total value of ΔL/L of 0.002, or 2000 µε. The breathing periodicity is ~1.4 ms, in agreement with the RTLS method. Finally, it can be seen that the total length change asymptotically relaxes to a value above the state before t0. Fitting a curve to guide the eye to this portion shows a final value of ~3 mm. While this is only a 10 ms snapshot of the total vessel circumference change, the result shows agreement with the OBR4600 results. The final state of the vessel circumference can again be attributed to either plastic deformation, or increased temperature, or some combination of these two variables.

#### 3.1.3. Comparison with Simulations

**Figure 21:**
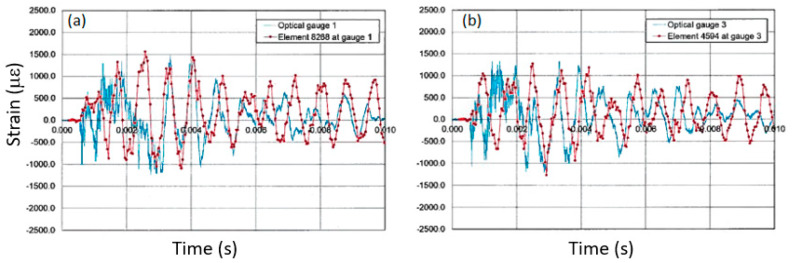
Comparisons between optical gauge and FE strains at (**a**) location 1 and (**b**) location 3 (see Figure 13 and Figure 15) for the 1.8 m vessel used in hydrotest 3685.

In the case of the 1.8 m vessel used for hydrotest 3685, as compared to the optical gauge measurements, the FE model accurately predicts the magnitude of the strains as seen in Figure 21. The figures show the raw experimental data (blue lines) overlaid with the FE prediction (red lines). The locations of the gauges were determined from pre-shot measurements of the gauges on the vessel surface. Figure 21a shows the results for gauge location 1 and Figure 21b shows the locations for gauge position 3 (see Figure 13 for reference).

The central goal of the FEA predictions is to show that the peak strain values and the primary breathing oscillation are predicted. Currently, improvements to the model are being made using the low noise and repeatable results from the optical diagnostics. The model itself is complex with the results heavily influenced by the shape of the vessel with the entrance and exit ports, the mass of all covers, and particularly the influence of the HE charge and any shielding contained within the vessel. Differences seen between the experimental results and the prediction can be attributed to all of these effects, but the results are showing a close match in the behaviors of most importance to qualifying the safety performance of the containment vessel-mainly the peak strain values and oscillation frequency.

**Figure 22:**
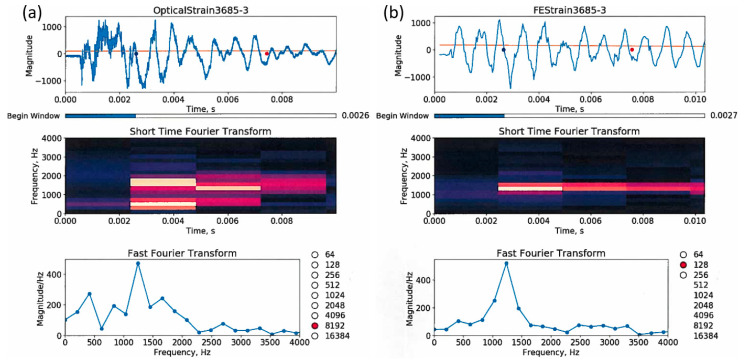
Comparisons between an optical strain measurement (**a**) and its corresponding FE computation (**b**) for gauge 3 on the 1.8 m vessel (see Figure 13). The top plots are the strain magnitude in micro-strains versus the time in seconds (note that between the plots, the x- and y-axis are scaled differently); STFTs of frequency in Hz versus time are plotted in the middle; and at the bottom are the FFTs between 2.6 and about 7.6 ms. From the optical strain data, the STFT and the FFT are computed using 2^13^ = 8192 data points; and from the FEA, we used 2^7^ = 128 points.

In addition to the strain amplitudes, an analysis of the vibration of the vessel was performed. Figure 22 shows the experimental data ((a) upper figure) and the FE prediction ((b) upper figure) for gauge location 3.An STFT was performed on the data using 8192 data points for the experimental data and 128 points for the FEA and can be seen in the middle plots of Figure 22.The results show similar spectral responses across the full temporal signal with a main peak around 1300 Hz. This is the calculated breathing mode for the vessel and appears once the vibrations including the mass of the lids and covers reaches a steady-state. An additional lower amplitude and frequency peak is seen under 500 Hz in the experimental results. It is unknown what the source of this is as it was not predicted in the FE. This is the subject for ongoing research. The STFT results also agree with the FFT seen in the lower plots in Figure 22. The FFT is applied to the time window from 2.6 to 7.6 ms. In both cases, the main frequency peak is found at 1300 Hz.

### 3.2. Overpressure Test (OPT)

#### RTLS Results

**Figure 23:**
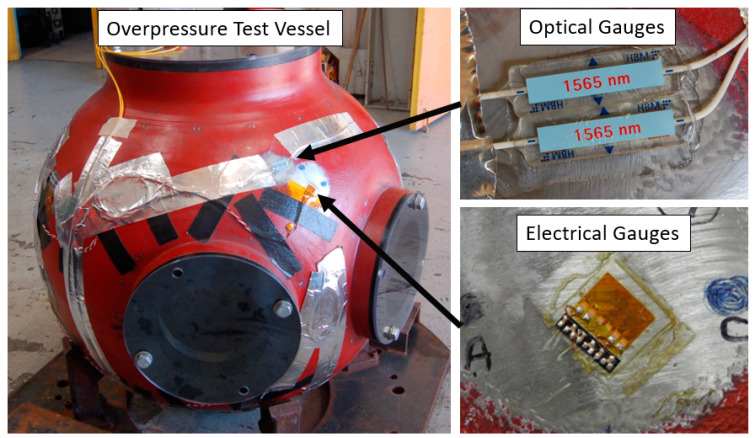
Optical gauges as placed on the overpressure test (OPT). Only plastic gauges were used for the optical measurements. A set of electrical gauges was also fielded which can be compared directly to the optical gauges.

A second experiment, an overpressure test or OPT was carried out on a 0.9 m vessel with only the RTLS techniques. This vessel test was different than hydrotest 3685 in that the vessel was a 0.9 m diameter vessel rather than the larger 1.8 m diameter vessel. The point of an OPT is to ensure that the vessel and any diagnostic feedthroughs in the vessel ports are able to withstand the pressures generated during the dynamic experiment. The 0.9 m vessels have a thinner wall of 2.82 cm nominal. The OPT consisted of 4 hand-packed cylinders of HE, in this case, composition 4 (C4) with a total mass of 0.277 kg per cylinder. However, the OPT also fielded a set of resistive electrical gauges on the surface. As the electrical gauges were a legacy diagnostic, they were primary and were given preference on the placement locations. The optical gauges were fielded nearby whenever possible so that direct comparisons could be made. Two identical chains of optical gauges of the plastic variety were fielded on the vessel for redundancy. Ensuring the optical gauges had the same orientation as well as being placed in the same locations was not always possible however so only a small subset of the optical and electrical gauges can be compared. Figure 23 shows an image of the optical gauges and electrical gauges as fielded on the vessel.

The gauge locations were chosen by the modelers as points of particular interest for measuring the strain on the vessel. The electrical gauges were applied to the surface first as they were the primary diagnostic for strain. The mounting technique used double bubble epoxy and the same mounting technique as described before. The optical gauges were then attached to the surface as close to the electrical gauges as possible. The optical gauges chosen for this experiment measured strain only along the FBG length while the electrical gauges chosen could measure in 3 directions. FBG gauges 3, 6, and 8 were fielded with locations and orientations very near the corresponding electrical gauges.

**Figure 24:**
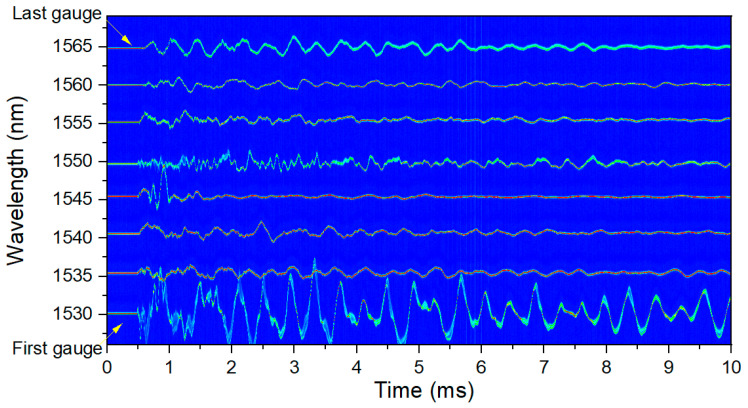
RTLS measurements for 8 plastic-backed gauges on the OPT vessel. The lowest trace corresponding to center wavelength 1530 nm was not fully adhered to the surface.

Figure 24 shows a spectrogram for an optical gauge chain on the OPT vessel. In the lowest trace, the strain measurements show crossings, indicating that the strain exceeded the amount expected for the design of our FBG chains. This gauge was partially peeled from the surface during movement of the vessel so it did not record real vessel strain data.

**Figure 25:**
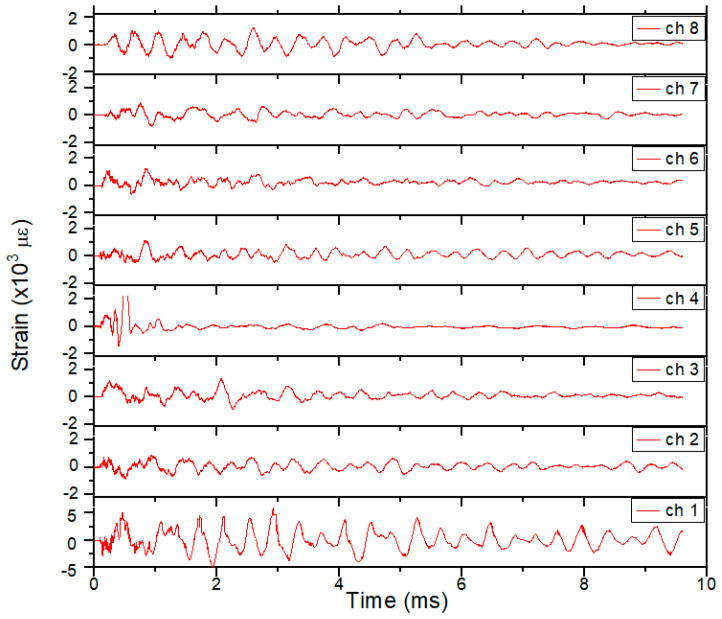
Extracted strain plots for each gauge fielded on the OPT. These correspond directly with the array seen in Figure 23.

The peak strain values as measured for all of the FBG gauges in a single chain (see Figure 25) are very close to those seen on hydrotest 3685 shown in Figure 18. 

**Figure 26:**
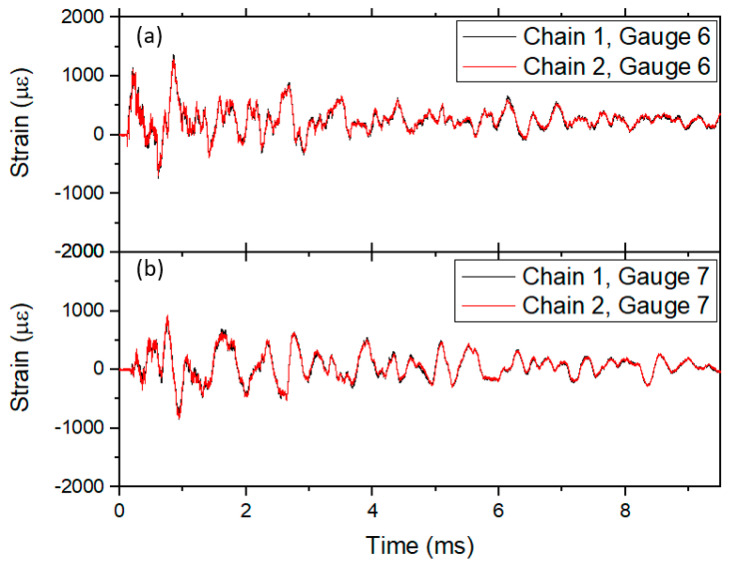
Strain plots for closely located optical gauges on the vessel. The gauges come from two distinct diagnostic chains (red and black lines) and were mounted as close as possible to each other. (**a**) Gauge 6 with wavelength 1555 nm and (**b**) gauge 7 with wavelength 1560 nm.

While the charge was smaller for the OPT, the 0.9 m vessel walls were also thinner, resulting in a comparable strain observed. The 8 independent measurements of the strain were obtained around the vessel with ringing periodicity of ~390 µs, again consistent with the FEA predicted vessel breathing after including the mass of the lids and covers. Two gauge chains were fielded and Figure 26 shows the agreement between two of the gauges. Many of the gauges on the vessel were not fully adhered to the surface but the agreement was still quite obvious. Gauges 6 and 7 as seen in Figure 26 showed the best agreement.

Maximum differences of ~7% of the strain are seen at certain times and can possibly be attributed to how the two gauges were bonded to the surface of the vessel but more likely to the small offset in position between the two gauges. The peak values were well below the maximum strain allowed for this particular vessel.

### 3.3. Comparison of Optical and Electrical Diagnostic with FEA Simulations

**Figure 27:**
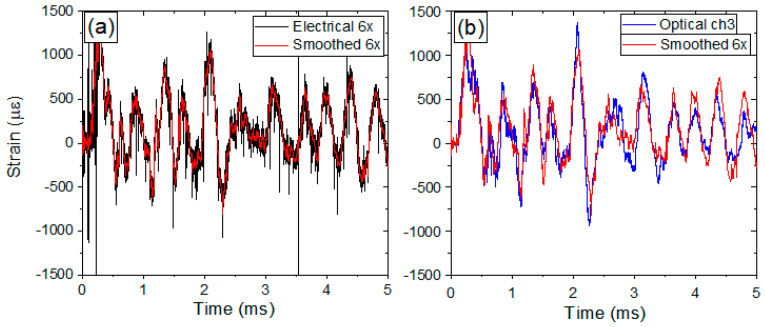
(**a**) Electrical gauge 6x raw (black line) and smoothed (red line). (**b**) Raw optical gauge (blue line) and smoothed electrical gauge 6x.

Due to vessel surface area and time constraints, the electrical and optical strain gauges were placed at various locations that were not always in close proximity or at the same orientation to each other. Since the purpose of this section is to show the differences between the optical and electrical gauges, three locations were chosen where the electrical and optical gauges were fielded under nearly identical conditions. Figure 12 shows the locations of these gauges as 3y, 6x, and 7x. Figure 27a shows the data for electrical gauge 6x after a 250 kHz low pass filter was implemented in software using a 53rd order finite impulse response filter. As the electrical gauges have long coaxial cables running between the diagnostic room and the firing point near the vessel, they are able to pick up a significant amount of electrical noise during the explosive test. This is seen in the figure as a high frequency signal remaining after filtering around 250 kHz which is much higher than any predicted strain (black curve). Additionally, spurious peaks of strain that exceed predictions by several times are also seen. To overcome even more noise in the data, the signal was further smoothed with 20 points adjacent averaging (red curve). This was done to see the overall strain amplitudes and slower periodic structure in the signals for a closer approximation to the optical signals. Figure 27b shows the optical data (blue curve) overlaid with the smoothed electrical signal (red curve). It should be emphasized that the optical data is in its raw form-no smoothing has been done to the temporal signal.

**Figure 28:**
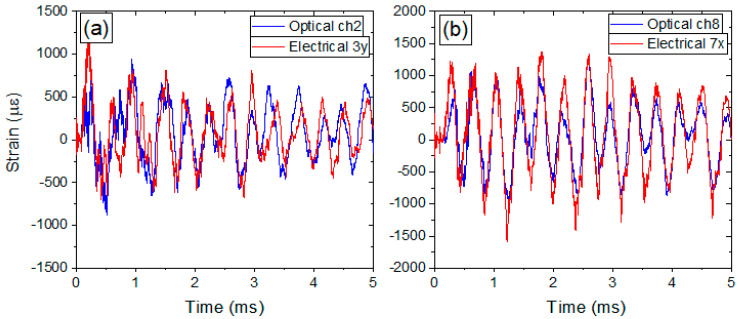
(**a**) Smoothed electrical gauge 3y raw (red line) and corresponding raw optical gauge (blue line). (**b**) Smoothed electrical gauge 7x raw (red line) and corresponding raw optical gauge (blue line).

As an optical diagnostic, the result shows none of the high frequency noise seen with the electrical gauge even though the optical diagnostic is sampled at nearly 2 MHz which is several times higher frequency than the noise seen with the electrical signals. To see the overall agreement between the legacy electrical gauges and the new optical gauges, the electrical signals will be smoothed with a 20 point adjacent average throughout this section. Figure 28 shows the results of the smoothed electrical gauges (red lines) plotted with the raw optical gauges (blue lines) for locations 3y and 7x. In all figures, the data for only the first 5 msareshown in order to demonstrate the agreement in the short timescale features of the strain. As can be seen in Figure 27b and Figure 28, the optical and electrical gauges show overall good agreement in terms of strain values, periodicity, and fine features in the temporal profiles. For a direct comparison between the electrical and optical data, the electrical data can only be justified to be smoothed by a ~3 point adjacent average since the data was sampled at 5 MHz as compared to the optical data at 1.7 MHz. However, a 20 pointtapboxcarfilter was used to most closely match the noise in the optical data. This demonstrates clearly the electrical gauges are highly sensitive to electrical noise. This was in addition to a 53rd order finite impulse response filter with an upper limit of 250 kHz applied to the raw signals. The largest source of this noise comes from the broad spectrum x-ray source used for radiography of the dynamic experiments. The electrical diagnostic picks up this noise while the optical diagnostic is not sensitive to it. While there is no physical basis for this degree of filtering, the optical gauges alleviate the issue of such significant post-processing of the raw signals. Differences in the measured strains seen in each plot can be attributed to small offsets in gauge positions on the surface of the vessel.

**Figure 29:**
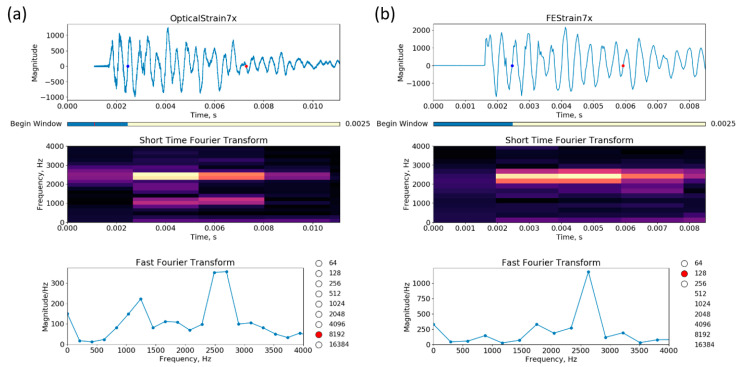
Optical strain measurement (**a**) versus its corresponding FE computation (**b**) for gauge 7x. On the plots, the x- and y-axis are scaled differently. The blue and red dots mark the beginning and end of the windows for the FFTs; with the optical gauge, the window contains 2^13^ = 8192 data points, and with the FE, 2^7^ = 128 data points.

Figure 29 shows the FE results for the 0.9 m vessel used in the OPT for a single optical gauge for comparison.

In the case of the 0.9 m vessel, the strain magnitude plots show that the FEA over-predicts the strain by a factor of two at the location of optical gauge 7x by nearly a factor of 2 (Figure 29 upper plots). We can see that we should add more damping to the FE model to reduce the vibration after about 7 ms. The STFTs (middle plots) are reasonably similar, and the FFT (lower plots), which is applied to the strain response between 2.5 and 6 ms, depicts a natural frequency of vibration at about 2600 Hz. Although the response is expected to be transient, it is interesting that for a few milliseconds the vessel is at a natural frequency, which produces the larger strains in the FEA and the test.

## 4. Discussions

Section 3.1.3 and Section 3.3 show the agreement between the experimental optical gauges and the FEA for a 1.8 m vessel and a 0.9 m vessel, respectively. Overall, the model predicts the strain amplitude well, particularly for the 1.8 m vessel. The frequency response for both vessels also agrees very well with the FEA. As was discussed previously, the RTLS diagnostics showed excellent agreement with the SITS and Luna diagnostics as fielded on hydrotest 3686. Peak values of localized strain, as recorded by the optical strain gauges, were recorded to be nearly 2000 µε. The total integrated strain around a single great circle of the vessel was also found to be a peak value of ~1.2 cm/5.7 m, or ~2100 µε. Additionally, the total vessel circumference over the course of 10 ms asymptotically approached a final increased length of ~3mm, consistent with the Luna measurements made at the same time. A length change of 3 mm/5.7 m corresponds to a strain of ~500 με. The RTLS measurements also show a final positive value for the local strain in the vessel surface of ~50–100 µε. Further work needs to be conducted to calibrate the gauges for use with such small values of strain.

The electrical gauges fielded on the OPT showed overall very good agreement with the optical gauges. As the optical gauges are not sensitive to electrical or RF noise, the signals were significantly cleaner that that observed with the electrical gauges. After significant smoothing of the electrical data, many features were shown to be consistent in the data, for example the ringing periodicity and the amplitudes of the strain. Many of the finer features were also represented in both diagnostics, although often with slightly different strain amplitudes. 

The inclusion of optical strain diagnostics on a containment vessel is a new diagnostic option for the Los Alamos National Lab hydrotest program. Due to the developmental nature of this work, a series of optical diagnostics was simultaneously fielded on the 3685 hydrotest as described in the preceding sections. It was found with the RTLS diagnostics that the lightweight plastic versions of the FBG strain gauges were significantly more reliable than the heavier metal-backed gauges. Properly welding the metal gauges may improve reliability but this is not an option as welding components onto the vessel is not allowed from a safety qualification standpoint. However, if the impulse on the vessel is expected to be small or the gauges can be ensured to remain adhered to the vessel, it is expected that the metal-backed gauges would respond equally to the plastic gauges. This would be valuable as the metal gauges are specifically designed to be far more robust than the plastic gauges and would survive in harsher environments.

The RTLS results were further verified through comparisons with two additional diagnostics. The first diagnostic fielded for verification of the RTLS results was the SITS technique. This diagnostic made a precise measurement of the total vessel circumference during the dynamic experiment. Peak strain values of ~2000 με were observed as determined from the total peak vessel circumference change of 1.2 cm out of 5.7 m. This was consistent with the localized peak strain measurements made with the RTLS technique. The ringing periodicity was found to be consistent between the RTLS and SITS techniques as 1.4 ms in agreement with the natural vessel breathing as seen in the FEA predictions. Additionally, the SITS technique showed that as the peak strain values integrated around the total circumference decayed, a final value above the initial state was measured. Further analysis of this final value is required however since SITS measurements ended 10 ms after the initiation of the experiment. Results indicate similar behaviors as compared with previous hydrotest experiments [15,16,17,18,19] when comparing the electrical diagnostic results. Hydrotest 3685 and the OPT are the first experiments showcasing the behavior of the optical diagnostics, and show excellent agreement with the electrical diagnostics although without the noise associated with the hydrotest experiments. The reduction in noise means the results can be compared directly to modeled predictions without the ambiguity associated with filtering the noisy data.

The second verification diagnostic was a series of nearly static vessel circumference length measurements made with a Luna OBR4600. The measurements showed a total length increase of 3.4 mm from before the experiment, consistent with the final result of the SITS technique. Over the course of an hour, this length did not change. However, an analysis of the thermal expansion of the vessel caused by the temperature increase from the dynamic experiment also showed an upper limit on the expected circumference change of 3.6 mm, consistent with the measured results. This showed that the increase could be fully attributed to thermal effects and the vessel was expected to relax back to its original circumference after the vessel fully cooled.

Finally, the results were also compared with legacy electrical strain gauges as fielded on an additional experiment. When the orientation and placement of the FBG and electrical gauges was consistent, the results showed excellent agreement. The large sources of noise inherent to hydrotests pose challenges to accurately predicting peak strains in the vessel. We have shown that the optical techniques show such a significant reduction in the noise, that no further smoothing is required to match the signals expected in the simulations.

All of the combined results showed good agreement with the FEA modeling. The overall peak values of strain and periodic oscillations were correctly reflected in the modeled predictions. The FEA modeling is currently being further improved using the results from the optical RTLS method on these two experiments. Further testing with simplified experimental parameters is being developed to improve the modeled predictions. The results implied that the vessels did not exceed the maximum strain that might compromise the vessel wall strength. With this evidence, the RTLS technique was demonstrated to be accurate for fast dynamic strain measurements even when high values of strain are expected. The reduced noise present in optical strain gauges versus electrical shows improved accuracy of the instantaneous strain measurement.

## Figures and Tables

**Table 1:** The techniques described in this paper with the strain types each is capable of measuring.

Diagnostic Name	Strain Type	Local vs. Global
RTLS	Elastic	Local
SITS	Elastic	Global
Luna-long fiber	Plastic	Global
Luna-short fiber	Plastic	Local
